# Detection of RUNX2 gene expression in cumulus cells in women undergoing controlled ovarian stimulation

**DOI:** 10.1186/1477-7827-10-99

**Published:** 2012-11-28

**Authors:** Myrto Papamentzelopoulou, Despina Mavrogianni, Vasiliki Dinopoulou, Haralampos Theofanakis, Fotodotis Malamas, Spyros Marinopoulos, Ritsa Bletsa, Elli Anagnostou, Kostas Kallianidis, Dimitris Loutradis

**Affiliations:** 1 Division of Human Reproduction, IVF Unit, 1st Department of Obstetrics and Gynaecology, Alexandra Hospital, Athens University Medical School, Athens, Greece

**Keywords:** RUNX2 expression, ART treatment, Cumulus cells, Estradiol levels, ICSI

## Abstract

**Background:**

RUNX2 is a transcription factor, whose expression has been recently identified in the mouse ovary. Regulation of RUNX2 expression and its function in the human ovary have not been determined yet. The aim of the present study is the investigation of the possible correlation between RUNX2 gene expression in cumulus cells and controlled ovarian stimulation and pregnancy outcomes after ART treatment.

**Methods:**

A total of 41 patients undergoing ICSI treatment for male factor infertility were enrolled into a specific ART program, during which cumulus cells were collected. The expression of RUNX2 gene in cumulus cells was examined by real-time PCR.

**Results:**

Concerning RUNX2 gene expression, 12 out of 41 women were detected with RUNX2 expression, with ratios ranging from 0.84 to 1.00, while 28 out of 41 women had no expression (ratio = 0). Only 1 woman presented a weak RUNX2 gene expression (ratio = 0.52). From 8 women that proceeded to pregnancy, 7 of them did not express RUNX2 gene in cumulus cells, while one was the woman with weak gene expression that also achieved pregnancy. The group of women without RUNX2 expression presented higher number of follicles (p = 0.013), higher number of retrieved oocytes (p = 0.016), higher basal LH serum levels (p = 0.016) and higher peak estradiol levels (p = 0.013), while the number of fertilized oocytes differed marginally between the two groups (p = 0.089). Moreover, RUNX2 expression was negatively associated with LH levels (OR = 0.22, p = 0.021) and E2 levels (OR = 0.25, p = 0.026).

**Conclusions:**

Consequently, based on the preliminary findings of the present pilot study a potential inhibitory mechanism of RUNX2 gene is observed in the ovary when high mRNA levels are detected, suggesting that RUNX2 could possibly be used as a candidate genetic marker in the monitoring of the outcome of an ART treatment.

## Background

It is well known that assisted reproductive technology (ART) treatment is a successful approach for infertile couples, which commonly overcomes the underlying infertility causes resulting in significantly higher pregnancy rates compared to natural conception. The variability in patient characteristics is directly related to the response to ART treatment, which consequently dictates the need for reliable, personalized diagnostic and therapeutic approaches to optimize efficacy, as well as safety outcomes. Significant scientific discoveries have made the causes of infertility more apprehensible and ART has facilitated the development of increasingly complex diagnostic tools, prognostic models and treatment options. As a result, it is crucial to extend investigation to more genetic factors, not necessarily related directly to the reproductive system, such as runt-related transcription factor 2 (RUNX2) gene, in order to conclude whether or not they are involved in the outcome of ART treatment [1].

RUNX2 (Cbfa1, AML-3, PEBP2αA) is well known for regulating both intramembranous and endochondral bone formation, as well as osteoblast development and differentiation and chondrocyte differentiation. It is a member of the runt family of transcription factors. The three mammalian RUNX proteins (RUNX1, RUNX2, RUNX3) share a highly conserved 128 amino acid DNA binding domain [2]. RUNX2 gene is located on chromosome 6, consists of eight coding exons and spans a genomic region of 130 kb. It contains a DNA-binding domain, a region of glutamine and alanine repeats in the N-terminal region and a region rich in proline-serine-threonine, which is necessary for transcriptional activation of target genes [3]. Moreover, RUNX2 association with the nuclear domain facilitates interaction with many co-regulatory proteins and chromatin-modifying complexes for the regulation of gene transcription [4].

In general, RUNX2 has been shown to play a crucial role in cell differentiation. Its expression has been recently identified in the rat ovary, but little is known about the regulatory mechanism of RUNX2 expression and the specific function of this protein in the human ovary. RUNX2 mRNA levels were shown to be increased by the luteinizing hormone (LH) surge in preovulatory follicles and newly forming corpus luteum in women and rodents, as determined by real time PCR, in situ hybridization, and human microarray analyses [5,6]. As a result, the LH-surge induced RUNX2 is functionally linked to various aspects of luteal development by regulating the expression of luteal specific genes. Moreover, in a model of doxycyline-inducible, triple transgenic mice (CMV-Cre;ROSA26 neoflox/+ − rtTA;Tet-O-RUNX2) highly induced RUNX2 transgene expression was observed in the ovary [7]. A recent study confirmed the strong association of RUNX2 gene with ovulation, luteinization and steroidogenesis, since RUNX2 was down-regulated in granulosa cells lacking (C/EBP)α and (C/EBP)β, transcriptional factors that are highly specialized in the ovulation process [8].

Concerning steroid effect on RUNX2 function, estradiol (E2) may enhance RUNX2 activity through direct interaction with estrogen receptor α (ER-α) without changing RUNX2 expression or DNA binding affinity, whereas glucocorticoids inhibit RUNX2 activity [9]. Specifically, estrogen may enhance RUNX2 activity in dose- and estrogen receptor-dependent ways regardless of changes in RUNX2 levels or its DNA binding potential. The stimulatory effect of estrogens on RUNX2 activity is lost when the DNA binding domain of the estrogen receptor is eliminated [10]. Moreover, RUNX2 induces aromatase expression, establishing a functional role in estrogen biosynthesis pathway. Unlike the stimulatory effect of estrogens and the inhibitory effect of glucocorticoids, androgens fail to increase RUNX2 activity, whereas RUNX2 strongly suppresses gene expression induced by all three steroids [11].

Based on recent findings of Park and her co-workers concerning RUNX2 detection in rat and human ovary [12], this pilot study focuses on RUNX2 gene expression in cumulus cells of human ovary, in order to examine its potential role in fertility and assisted reproduction technology treatment outcome. Specifically, RUNX2 expression in cumulus cells of women enrolled into an ART program was correlated with biochemical, clinical and ovarian stimulation factors with an upper aim to conclude whether and to what extent RUNX2 is involved in the controlled ovarian stimulation and pregnancy outcome.

## Methods

### Patient population

41 patients were recruited into a specific ART protocol including intracytoplasmic sperm injection (ICSI) treatment [13] for male factor infertility. All women were pre-menopausal, 25–45 years of age with a normal hormonal profile according to WHO guidelines. Each of them had at least one unsuccessful ICSI cycle in the past, but had not received ovulation induction or other hormonal treatment within three months preceding the study.

Patients’ demographic characteristics (age, BMI, duration of infertility) were recorded before ovarian stimulation protocol. In addition, stimulation dose, duration of ovarian stimulation, number of follicles, oocytes and fertilized oocytes were determined for each patient during treatment cycle. It should be noted that day 2 hormonal profile consisting of follicle-stimulating hormone (FSH), LH and prolactin (PRL) had been measured within the previous six months. On the other hand, estrogen levels were being recorded throughout ovarian stimulation protocol.

### Ovulation induction

Soon after the design of the study and before patient recruitment, the protocol was approved by the Ethics Committee of Alexandra Hospital and a signed informed consent was obtained from each participant of this study.

All patients underwent long luteal GnRH-agonist down-regulation protocol. A baseline ultrasound scan on day 21 of the preceding cycle was followed by intranasal Buserelin spray (Superfact; Hoechst, Frankfurt, Germany) initiation at a dose of 100 μg five times daily for 14 days. Pituitary down-regulation and subsequent ovarian suppression were confirmed with ultrasound scan (absence of ovarian cysts and endometrial proliferation) and low serum E2 levels (<40 pg/ml). If the above criteria were not met, down-regulation was extended for another week. As soon as pituitary desensitization had occurred, a fixed 5-day pretreatment with 200 IU/day of rLH (Luveris; Serono, Geneva, Switzerland) followed by stimulation with a fixed dose of 225 IU/day of rFSH (Gonal-F; Serono, Geneva, Switzerland) for five days and adjustment of the dose of rFSH thereafter was applied. Serum E2 levels were measured on day 5 and daily from day 8 of rFSH stimulation. Ultrasound scan was performed on a daily basis from day 9 and both follicular growth and endometrial thickness were recorded.

A dose of 10,000 IU of hCG (Pregnyl; N.V. Organon, Oss, Netherlands), triggering final oocyte maturation, was administered once the mean diameter of at least two follicles was >18 mm and serum E2 was rising. Oocytes were retrieved by transvaginal ultrasound-guided ovarian puncture 35–36 h post hCG injection. Oocyte maturation was assessed under the microscope following stripping of the cumulus-oocyte complexes and mature oocytes (metaphase II) were used for ICSI. On day 3, embryo transfer took place, with 3 embryos per cycle being transferred back to the uterus and 2,500 IU of hCG were given on the days of embryo transfer and four days later for luteal phase support. Pregnancy was confirmed with detection of serum hCG levels 14 days following egg collection, whereas clinical pregnancy was defined as a gestational sac with positive fetal heart activity seen on transvaginal ultrasound scan two weeks later.

### RNA extraction and cDNA preparation

In order to determine RUNX2 mRNA expression, cumulus cells were collected during oocyte retrieval. The cells were segregated from cumulus oocyte complexes (COCs) through the process of stripping using hyaluronidase. COCs from approximately 5 oocytes per woman were pooled. RNA was extracted using the RNeasy Micro Kit (Qiagen, Valencia, CA, USA). RNA extraction was performed as previously described [14]. The extracted RNA was a product of cumulus cells pooled from several COCs and not only from the oocytes that proceeded to embryo transfer. Moreover, RNA concentration of each sample was determined by spectrophotometry and its quality was evaluated by agarose gel electrophoresis.

cDNA preparation was performed using 20 ng of total RNA. RNA was reverse-transcribed using 0.5 mM dNTP mix (Ambion, Austin, Tx, USA), 5 μM oligo dT Primer (Ambion, Austin, Tx, USA), 1xRT buffer (Ambion, Austin, Tx, USA), 80 U ribonuclease inhibitor (Invitrogen Life Technologies), 1600 U M-MLV reverse transcriptase (Invitrogen Life Technologies) and nuclease free water (Ambion, Austin, Tx, USA) to a total volume of 40 μl. The reactions were carried out in Mastercycler (Eppendorf) with the following conditions: 80°C for 3 min, 42°C for 60 min and 92°C for 10 min. The resulting cDNAs were stored at −20°C.

### Real-time PCR

RUNX2 gene expression in cumulus cells was examined by real-time PCR. The oligonucleotide primers and probes for RUNX2 gene were synthesized by TIB MOLBIOL [GenBank accession no. NM_001024630]. Primers used for RUNX2 gene were checked using the BLAST program and the template sequence was shown to be of 280 bp length. A PCR using the RUNX2 primers 4was applied and the PCR products were run in an agarose gel to verify the 280 bp expected length.

G6PDH was used as a control gene. In all experiments a no template control was used. Real-time PCR was performed on LightCycler 480 II (Roche) with the following parameters: one cycle at 95°C for 10 min for pre-incubation and 40 cycles for amplification [95°C for 10 sec, 55°C for 20 sec, 72°C for 10 sec]. The emitted fluorescence was detected at 640 nm. RUNX2 PCR mixtures contained 5 μl cDNA, 5x master mix (LightCycler 480 Genotyping Master, Roche), 20 μM of each primer, 20 μM of each probe and PCR-grade water (LightCycler 480 Genotyping Master, Roche) to a total volume of 20 μl. G6PDH PCR mixtures contained 5 μl G6PDH standard (LightMix Kit human G6PDH, TIB MOLBIOL), 5x master mix (LightCycler 480 Genotyping Master, Roche), 16x G6PDH (LightMix Kit human G6PDH, TIB MOLBIOL), 25 mM MgCl_2_ (LightCycler 480 Genotyping Master, Roche) and PCR-grade water (LightCycler 480 Genotyping Master, Roche) to a total volume of 20 μl.

Results were obtained as a Cp-value and number of copies. Cp-value represents the time-point at which the fluorescence of the sample rises above the background fluorescence during the real-time PCR process. RUNX2 gene results were normalized to G6PDH results based on the reference of Müller and his co-workers [15]. Serial dilutions of G6PDH gene were performed to obtain a standard curve, as well as to verify the sensitivity of the experiment.

### Statistical analysis

The results of the present study were statistically analyzed using SPSS package program. Due to the deviation from normality, non-parametric Mann–Whitney test was applied in order to evaluate the univariate association of demographic and biochemical factors, as well as factors included in the ovarian stimulation profile of each patient and RUNX2 gene expression in cumulus cells. In addition, multiple logistic regression was performed to investigate possible determinants of RUNX2 gene expression. It should be noted that statistical significance was defined at the level of 5% (p < 0.05).

## Results

### RUNX2 gene expression in cumulus cells

**Figure 1:**
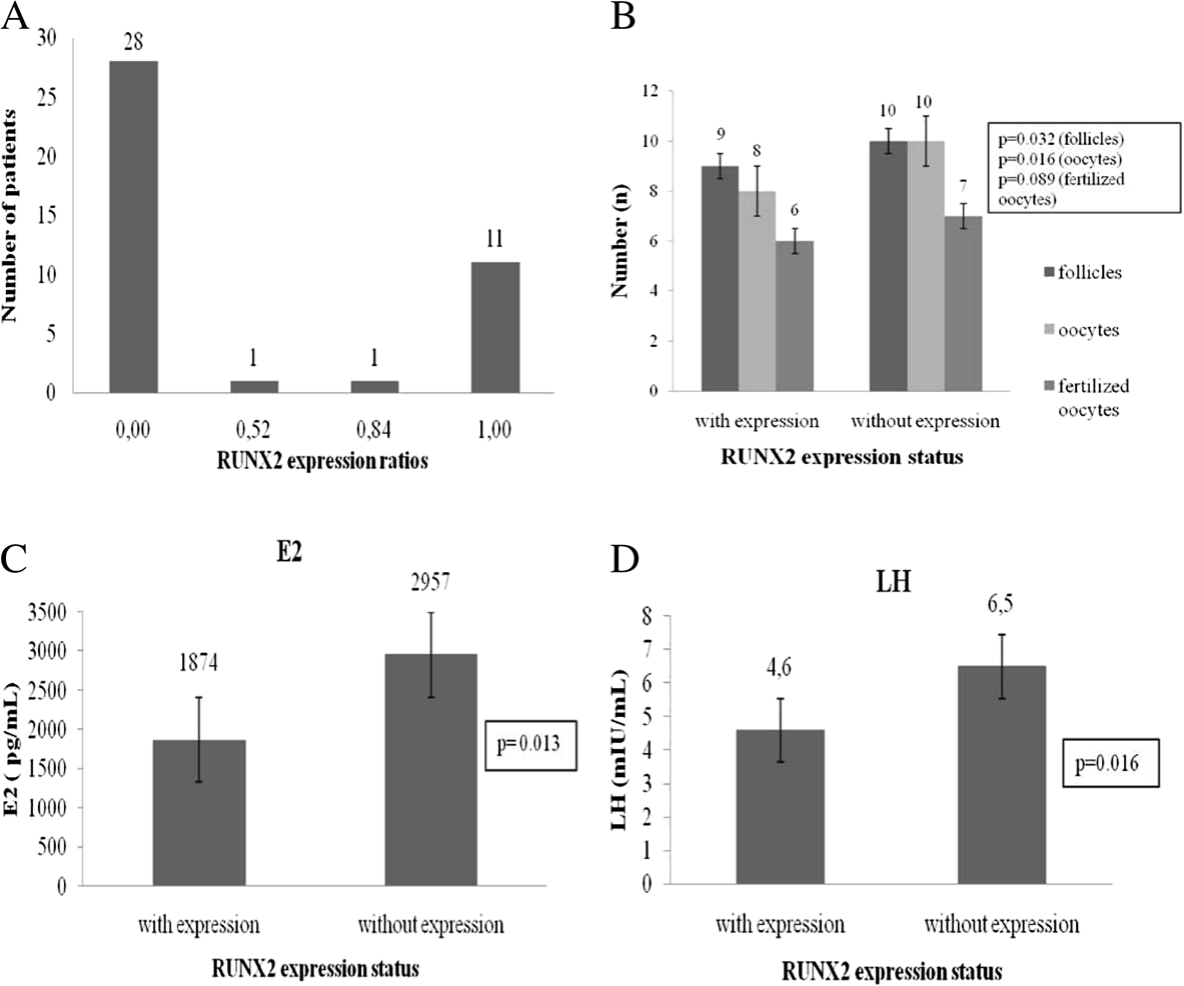
**Distribution of RUNX2 gene expression (A) on patient’s population and statistically significant correlations between ovulation induction factors, such as number of follicles, oocytes, fertilized oocytes (B) and hormone serum levels for E2 (C) and LH (D) respectively. **RUNX2 gene expression was determined by real-time PCR for 41 patients and categorized into two groups of patients, with expression and without expression. Expression ratios (RUNX2 copies/G6PDH copies) were used for the evaluation of RUNX2 gene expression.

In order to determine the expression of RUNX2 gene in cumulus cells, samples were isolated from 41 women that participated into a specific protocol of artificial reproduction technology. The samples underwent RNA extraction and cDNA synthesis and RUNX2 gene expression in cumulus cells was studied using quantitative PCR (real- time PCR). The expression results are presented herein as ratios of RUNX2/G6PDH expression, resulting to ratios varying from 0 to 1. Figure 1A illustrates the distribution of RUNX2 gene expression on patient’s population. In particular, RUNX2 expression was detected in 12 out of 41 women, with ratios ranging from 0.84 to 1.00, while 28 out of 41 women had no expression (ratio = 0). Only 1 woman presented a weak RUNX2 gene expression (ratio = 0.52). From 8 women that proceeded to pregnancy, 7 of them did not express RUNX2 gene in cumulus cells, while one was the woman with weak gene expression that also achieved pregnancy.

The non-parametric Mann–Whitney test was applied to compare the clinical, biochemical and ovarian stimulation factors in the two groups of patients depending on RUNX2 gene expression (Table 1). At this point, it should be clarified that there was no statistically significant difference between women with (n = 13) and without expression (n = 28) regarding age (p = 0.886) and BMI (p = 0.320). The groups of women with and without RUNX2 expression differed at a statistically significant degree in the number of follicles (p = 0.032), the number of oocytes (p = 0.016), as well as in the LH levels (p = 0.016). More specifically, women without RUNX2 expression had higher LH levels (6.5 mIU/ml versus 4.6 mIU/ml, p = 0.016) and higher numbers of follicles (10 vs 9, p = 0.032) and oocytes (10 vs 8, p = 0.016) compared to women that expressed RUNX2 in cumulus cells. Concerning the estradiol levels at the day of hCG administration, women without RUNX2 expression had higher levels (2957 pg/ml versus 1874 pg/ml, p = 0.013). Moreover, the two groups differed marginally at the 10% significance level in the number of fertilized oocytes (p = 0.089). The non-parametric correlation between RUNX2 and estradiol is statistically significant and indicates negative association (r = −0.40, p = 0.010). Figures 1 B, C and D demonstrate the statistically significant correlations between RUNX2 expression status and ovarian stimulation related factors, such as number of follicles, oocytes, fertilized oocytes, as well as LH and serum E2 levels. Moreover, comparing RUNX2 expression in the pregnant and the non-pregnant women, no statistically significant difference was observed, as indicated by Fisher’s exact test (p = 0.398), which was used for the categorical transformation of the original values into the two distinct groups, particularly with and without expression groups, as previously mentioned.

**Table 1:** RUNX2 expression status in cumulus cells in correspondence with biochemical/ovarian stimulation factors and pregnancy achievement

	**With expression (n = 13) Median ± SD**	**Without expression (n = 28) Median ± SD**	**p-value**
*Age (yrs)*	32.0 ± 5.2	34.5 ± 4.0	0.877
*BMI (kg/m*^*2*^*)*	24.2 ± 6.6	22.7 ± 3.2	0.320
*17.9-25.0*	53.8%	71.4%	
*25.1-30.0*	23.1%	21.4%	
*30.1-42.8*	23.1%	7.1%	
*Infertility (yrs)*	7.0 ± 4.0	4.0 ± 3.0	0.129
*FSH (mIU/ml)*	6.4 ± 2.6	6.4 ± 2.7	0.966
*LH (mIU/ml)*	4.6 ± 1.6	6.5 ± 2.7	0.016
*PRL (ng/ml)*	12.9 ± 9.5	15.9 ± 5.7	0.742
*Stimulation dose (IU)*	2600 ± 1446	2138 ± 898	0.165
*Ovarian stimulation (days)*	10.0 ± 1.0	10.0 ± 3.0	0.167
*E2 levels at the day of hCG (pg/ml)*	1874 ± 700	2957 ± 1543	0.013
*Number of follicles*	9.0 ± 1.0	10.0 ± 3.0	0.032
*Number of oocytes*	8.0 ± 1.0	10.0 ± 2.0	0.016
*Number of fertilized oocytes*	6.0 ± 2.0	7.0 ± 3.0	0.089
*Oocyte maturity rate*	0.75 ± 0.1	0.73 ± 0.1	0.817
*Good quality embryos*	0.69 (n = 9)	0.79 (n = 22)	0.465
*Clinical pregnancy rate*	0.12 (n = 1)	0.25 (n = 7)	0.210

Distribution of age, biochemical and ovarian stimulation factors by RUNX2 expression in cumulus cells (Mann–Whitney test), as well as oocyte maturity and clinical pregnancy rates and good quality embryos corresponded to RUNX2 expression status. Statistical significance was defined at the level of 5% (p < 0.05).

On the other hand, Table 2 presents multiple logistic regression derived ORs for 95% confidence intervals (CIs) for the correlation between RUNX2 gene expression in cumulus cells, age, number of follicles and oocytes, as well as LH and E2 levels. As presented, based on age adjusted analysis, the number of follicles and oocytes are negatively associated with RUNX2 expression, a finding which is significant at the 10% level (p = 0.051 and p = 0.062 respectively). Furthermore, age adjusted analysis revealed a negative statistically significant correlation between estradiol levels and RUNX2 expression at the 5% significance level (p = 0.026). In addition, a persistent negative statistically significant association between RUNX2 expression and LH levels is observed (p = 0.021). Specifically, for one standard deviation increase in LH levels the odds ratio for RUNX2 expression is 0.22 (0.06-0.80).

**Table 2:** Correlations between RUNX2 gene expression, age and ovarian stimulation factors

	**Crude**		**Adjusted to age**		**Mutually adjusted**	
	**OR (95%CI)**	**p-value**	**OR (95%CI)**	**p-value**	**OR (95%CI)**	**p-value**
*Age (yrs)*	1.1 (1.45-2.66)	0.851	-	-	0.76 (0.26-2.32)	0.619
*Number of follicles*	0.39 (0.15-1.01)	0.051	0.37 (0.13-1.01)	0.051	0.44 (0.16-1.23)	0.117
*Number of oocytes*	0.34 (0.11-1.07)	0.065	0.31 (0.09-1.06)	0.062	-	-
*LH (mIU/ml)*	0.23 (0.06-0.80)	0.021	0.22 (0.06-0.80)	0.021	0.24 (0.06-0.89)	0.033
*Ε2 (per SD)*	0.28 (0.09-0.89)	0.031	0.25 (0.08-0.85)	0.026	0.32 (0.10-1.03)	0.055

Multiple logistic regression with the derived odds ratios (ORs) for 95% confidence intervals (CIs) for the association between RUNX2 gene expression in cumulus cells, age, number of follicles and oocytes, as well as LH and E2 levels.

## Discussion

The aim of the present pilot study was to determine the expression of RUNX2 gene in cumulus cells of women undergoing a certain protocol of assisted reproduction and to investigate its possible correlation with the controlled ovarian stimulation and pregnancy outcome. RUNX2 is known to be an essential factor for chondrocyte and osteoblast differentiation and bone formation. However, the expression of this gene in non- skeletal tissues and cells, as reported herein, may indicate other important functions of RUNX2. Specifically, in a recent study, RUNX2 gene expression was detected in periovulatory granulosa cells of rat and human ovaries, while a significant up-regulation of RUNX2 gene expression within 12 h after pre-treatment with hCG was revealed [12]. Results obtained from our study demonstrated RUNX2 gene expression in cumulus cells of 13 out of 41 women that participated in the ART program with expression ratios ranging from 0.52 to 1.00. One possible interpretation of such RUNX2 mRNA levels could be the hCG administration performed in the present ART protocol, a finding that agrees with the previously reported results of Park and her co-workers, since hCG induced up-regulation of RUNX2 in rat preovulatory granulosa cells [12]. On the other hand, it is of great interest that those women did not proceed to pregnancy, except for one woman with a weak RUNX2 gene expression that achieved pregnancy. Although hCG administration generally increases RUNX2 expression, RUNX2 absence in the other 28 women is remarkable. This finding could be attributed to the combination of hCG and rLH/rFSH administration, which may lead to inhibitory activities in the ovulation process of those women lacking RUNX2 gene expression. In the previously mentioned study of Park [12], rats were treated with pregnant mare serum gonadotropin (PMSG) and hCG to induce follicular development and ovulation, respectively, whereas in our study women were pre-treated with rLH and rFSH and administered with hCG. Moreover, in our study RUNX2 expression was studied at a specific time of the ART protocol, not having information about the expression profile of RUNX2 the previous days of the ovulation induction.

Nevertheless, it should be noted that a generally high expression profile of RUNX2 gene has been detected in periovulatory follicles, newly forming corpora lutea and corpora lutea from previous estrous cycles in rat ovary [16]. In addition, a previous study revealed that the LH-surge induced RUNX2 expression is functionally linked to various aspects of luteal development by regulating the expression of specific luteal genes [6]. Recent data outlined the antagonistic role of RUNX2 in regulating periovulatory gene expression. More specifically, RUNX2 up-regulates the expression of luteal genes, such asRgc32, Mmp13, Ptgds, Fabp6 and Abcb1a, whereas down-regulates the transcription of specific ovulatory genes in luteinizing granulosa cells [12,17].

An interesting observation in our study concerns the higher number of follicles and retrieved oocytes in the group of women without RUNX2 gene expression, as well as higher serum LH levels. These findings suggest a possible inhibitory mechanism of RUNX2 in different pathways involved in oocyte maturation. It is well documented that RUNX2, as a transcription regulator of cell differentiation and proliferation, is involved in many biochemical pathways. A recent study revealed the transcriptional induction of hyaluronan and proteoglycan link protein 1 (Hapln1) gene expression by RUNX2 activity, through endogenous RUNX2 binding to the Hapln1 promoter region [18]. It should be mentioned that Hapln1 enhances COC expansion by acting as a stabilizer of the cumulus matrix, thus promoting a successful ovulation [19]. However, the present data correlate RUNX2 expression in cumulus cells with a lower number of follicles and retrieved oocytes. It could be suggested that the interaction Hapln1-RUNX2 is time-specific. RUNX2 expression was studied on the day of oocyte collection. Probably a daily study of expression after hCG administration and before oocyte collection may reveal that RUNX2-HalpnI is expressed until cumulus cells matrix is expanded and then declines acting as an activation mechanism for the rest of the procedure. Furthermore, in rat ovary Hapln1 expression was affected by both RUNX1 and RUNX2 expression [18], indicating the need for further investigation of RUNX2-RUNX1 interaction in human cumulus cells. Interestingly, RUNX1 transcription was found to be suppressed directly by RUNX2 over-expression in preovulatory granulosa cells, providing another insight of RUNX genes involvement in ovulation [17].

Regarding E2 levels at the day of hCG administration, several studies in the literature confirm that E2 levels play a fundamental role in cytoplasmic maturity, as well as in the quality of the embryos [20]. The way estrogens affect RUNX2 activity as well as RUNX2-ER-α interactions are both complex. ER-α forms dimers with RUNX2 and the stimulatory effect of estrogens on RUNX2 activity is lost when the DNA binding domain of the estrogen receptor is eliminated. In cultured osteoblasts estrogen enhances RUNX2 activity in dose and estrogen-receptor dependent ways without changes in total RUNX2 levels or its affinity for DNA. On the other hand, in COS7 cells E2-bound ER-α suppresses the transactivation activity of RUNX2. Furthermore, E2 inhibits RUNX2 in late osteoblast cultures, while in early cultures RUNX2 may be stimulated by E2 [21]. ER-α interacts with RUNX2 through multiple domains and the repression occurs independently of the activation of estrogen response element containing genes. RUNX2 suppresses estrogen activity by decreasing the effect of estradiol on reporter gene expression driven by the estrogen receptor response element [10]. Recent studies on breast cancer cells disclosed that ER-α physically binds RUNX2 and inhibits expression of several RUNX2 target genes, providing a strong antagonistic correlation between the two genes in a different cellular type [22,23]. This opposing effect is verified by our results. The non-parametric correlation between RUNX2 and E2 is statistically significant and indicates negative association (r = −0.40, p = 0.010). Comparing E2 levels between women with and without RUNX2 expression, women without expression present higher levels to a statistically significant degree (2957 pg/ml versus 1874 pg/ml, p = 0.013). An explanation of higher E2 levels in the group without RUNX2 expression in our study could be the down–regulation phenomenon in RUNX2 expression that may occur in this group of patients. Since E2-bound ER-α suppresses RUNX2 in a strong and specific mechanism, additional changes in ER-α expression between groups, apart from the different E2 levels, could also possibly affect RUNX2 expression, although ER-α has not been investigated in our study.

## Conclusions

In summary, even though RUNX2 remains a less studied protein for its association with controlled ovarian stimulation outcome in ART treatment, a possible correlation of RUNX2 gene expression in cumulus cells and ovarian stimulation factors is observed in our study. It is remarkable that the detectable gene expression is not associated with a favorable outcome. The possible interaction of this transcription factor with different pathways involved in ovulation and implantation needs further investigation to confirm its inhibitory effect.

## Competing interests

The authors declare that they have no competing interests.

## Authors’ contributions

MP analyzed RUNX2 expression by real-time PCR and wrote first draft. DM set up real-time PCR, supervised MP. VD and HT collected cumulus cells. RB collected cumulus cells and cultured embryos. SM performed the statistical analysis. FM and EA completed writing of paper. KK gained approvals, performed U/S scans and retrieved oocytes. DL led research programme, supervised researchers and completed writing of paper. All authors read and approved the final manuscript.

## References

[B1] Devroey P Fauser BCJM Diedrich K Approaches to improve the diagnosis and management of infertility Hum Reprod Update 2009 15 4 391 408 10.1093/humupd/dmp012 19380415 PMC2691653

[B2] Stock M Schafer H Fliegauf M Otto F Identification of novel target genes of the bone-specific transcription factor RUNX2 J Bone Miner Res 2004 19 959 972 10.1359/jbmr.2004.19.6.959 15190888

[B3] Wang GX Sun RP Song FL A novel RUNX2 mutation (T420I) in Chinese patients with cleidocranial dysplasia Genet Mol Res 2010 9 1 41 47 10.4238/vol9-1gmr685 20082269

[B4] Lou Y Javed A Hussain S Colby J Frederick D Pratap J Xie R Gaur T Wijnen AJ Jones SN A RUNX2 threshold for the cleidocranial dysplasia phenotype Hum Mol Genet 2009 18 3 556 568 19028669 10.1093/hmg/ddn383 PMC2638795

[B5] Hernandez-Gonzalez I Gonzalez-Robayna I Shimada M Wayne CM Ochsner SA White L Richards JS Gene expression profiles of cumulus cell oocyte complexes during ovulation reveal cumulus cells express neuronal and immune-related genes: does this expand their role in the ovulation process? Mol Endocrinol 2006 20 6 1300 1321 16455817 10.1210/me.2005-0420

[B6] Park ES Choi S Lind AK Dahm-Kahler P Brannstrom M Carletti M Christenson KL Curry TE Jo M The LH surge-induced RUNX2 transcription factor regulates the expression of specific genes in luteinizing granulosa cells Biol Reprod 2009 81 357

[B7] He N Xiao Z Yin T Stubbs J Li L Quarles LD Inducible expression of RUNX2 results in multiorgan abnormalities in mice J Cell Biochem 2011 112 2 653 665 10.1002/jcb.22968 21268087 PMC5079519

[B8] Fan HY Liu Z Johnson PF Richards JS CCAAT/enhancer-binding proteins (C/EBP)-α and -β are essential for ovulation, luteinization, and the expression of key target genes Mol Endocrinol 2011 25 2 253 268 10.1210/me.2010-0318 21177758 PMC3386543

[B9] Alarcón-Riquelme ME Role of RUNX in autoimmune diseases linking rheumatoid arthritis, psoriasis and lupus Arthritis Res Ther 2004 6 169 173 10.1186/ar1203 15225361 PMC464920

[B10] McCarthy TL Chang WZ Liu Y Centrella M RUNX2 integrates estrogen activity in osteoblasts J Biol Chem 2003 278 44 43121 43129 10.1074/jbc.M306531200 12951324

[B11] Jeong JH Jung JK Kim HJ Jin JS Kim HN Kang SM Kim SY Wijnen AJ Stein JL Lian JB The gene for aromatase, a rate-limiting enzyme for local estrogen biosynthesis, is a downstream target gene of RUNX2 in skeletal tissues Mol Cell Biol 2010 30 10 2365 2375 10.1128/MCB.00672-09 20231365 PMC2863706

[B12] Park ES Lind AK Dahm-Kähler P Brännström M Carletti MZ Christenson LK Curry TE Jo M RUNX2 transcription factor regulates gene expression in luteinizing granulosa cells of rat ovaries Mol Endocrinol 2010 24 4 846 858 10.1210/me.2009-0392 20197312 PMC2852356

[B13] Palermo G Joris H Devroey P Van Steirteghem AC Pregnancies after intracytoplasmic injection of single spermatozoon into an oocyte Lancet 1992 340 8810 17 18 10.1016/0140-6736(92)92425-F 1351601

[B14] Patsoula E Loutradis D Drakakis P Michalas L Bletsa R Michalas S Messenger RNA expression for the follicle-stimulating hormone receptor and luteinizing hormone receptor in human oocytes and preimplantation-stage embryos Fertil Steril 2003 79 5 1187 1193 10.1016/S0015-0282(03)00071-2 12738515

[B15] Müller MC Saglio G Lin F Pfeifer H Press RD Tubbs RR Paschka P Gottardi E O’Brien SG Ottmann OG An international study to standardize the detection and quantitation of BCR-ABL transcripts from stabilized peripheral blood preparations by quantitative RT-PCR Haematologica 2007 92 970 973 10.3324/haematol.11172 17606448

[B16] Choi S Park ES Jo M The expression pattern of core binding factor during the periovulatory period in the rat ovary Biol Reprod 2007 77 97 c–98

[B17] Park ES Park J Franceschi RT Jo M The role for runt related transcription factor 2 (RUNX2) as a transcriptional repressor in luteinizing granulosa cells Mol Cell Endocrinol 2012 362 1–2 165 175 22713854 10.1016/j.mce.2012.06.005 PMC3864655

[B18] Liu J Park ES Curry TE Jo M Periovulatory expression of hyaluronan and proteoglycan link protein 1 (Hapln1) in the rat ovary: hormonal regulation and potential function Mol Endocrinol 2010 24 1203 1217 10.1210/me.2009-0325 20339004 PMC2875804

[B19] Sun GW Kobayashi H Suzuki M Kanayama N Terao T Link protein as an enhancer of cumulus cell-oocyte complex expansion Mol Reprod Dev 2002 63 223 231 10.1002/mrd.90008 12203832

[B20] Loutradis D Drakakis P Kallianidis K Milingos S Dendrinos S Michalas S Oocyte morphology correlates with embryo quality and pregnancy rate after intracytoplasmic sperm injection Fertil Steril 1999 72 2 240 244 10.1016/S0015-0282(99)00233-2 10438988

[B21] Khalid O Baniwal SK Purcell DJ Leclerc N Gabet Y Stallcup MR Coetzee GA Frenkel B Modulation of RUNX2 activity by estrogen receptor-alpha: implications for osteoporosis and breast cancer Endocrinology 2008 149 12 5984 5995 10.1210/en.2008-0680 18755791 PMC2613062

[B22] Chimge NO Baniwal SK Little GH Chen YB Kahn M Tripathy D Borok Z Frenkel B Regulation of breast cancer metastasis by Runx2 and estrogen signaling: the role of SNAI2 Breast Cancer Res 2011 13 6 R127 10.1186/bcr3073 22151997 PMC3326569

[B23] Chimge NO Baniwal SK Luo J Coetzee S Khalid O Berman BP Tripathy D Ellis MJ Frenkel B Opposing effects of Runx2 and estradiol on breast cancer cell proliferation: in vitro identification of reciprocally regulated gene signature related to clinical letrozole responsiveness Clin Cancer Res 2012 18 3 901 911 10.1158/1078-0432.CCR-11-1530 22147940 PMC3277803

